# Annotations

**Published:** 1903-07-18

**Authors:** 


### ANNOTATIONS.

### The Open Board System.

At one time, when education was less general than at present, hospital government by open board was much in vogue. The idea underlying this system is that every governor of the hospital shall have the right to attend every meeting in the board-room at his will and pleasure, and so take part in the management of the hospital. In theory it was an open democracy; in practice, whenever party feeling prevailed upon any subject within the hospital walls, it has been made the means of enforcing the will of the oldest and often the least capable governors, to the injury of the best interests of the hospital. Almost everywhere this system has been abolished in favour of a responsible committee of management, consisting of the most enlightened and energetic governors of the institution. At Northampton, when a new hospital had to be built, the large contributors made it a condition that the open board should be abolished, and this step is to be taken at a meeting of the governors on the 18th instant. In London, St. George's Hospital is rapidly becoming discredited with the public and financially unsound for want of income, mainly because the open board system is still persisted in there. If St. George's Hospital is ever to prosper, as it ought to prosper, it is essential that those who may be asked to provide the money, which it at present needs for building purposes, shall make their gifts conditional upon the abolition of the open board. Until St. George's Hospital has a responsible board of administration consisting mainly of business men, the best informed and most liberal contributors to hospitals are not likely to place it on the list of those institutions to which they habitually give. If Northampton can thus exhibit the effects of education and experience in hospital matters, it is surely not too much to hope, that at least equal enlightenment may be displayed in the neighbourhood of Hyde Park Corner, the very centre of the metropolis of the Empire.

### Parents and Plain Speaking.

One of the questions which must trouble thinking and conscientious parents as their boys and girls approach maturity is that of the amount of knowledge which they ought to have regarding the laws of sex. It is difficult to fix the time when the unthinking ignorance of childhood should be exchanged for the knowledge which men and women ought to possess, and the common mode of procedure is simply to shelve the subject altogether. Whether this would be wise, even if the silence thus maintained between parents and children meant that the latter really knew nothing of the laws of their being, is doubtful, but assuredly they will learn sooner or later, and in all probability from teachers who will show scant reverence for them. There is always at hand a companion of wider experience—in all probability illegitimately gained—to whisper as a prurient secret what should be realised as a simple fact. In many cases the knowledge thus gained will be tested by the learner, with results to soul and body which are only too well known. The question is a difficult one, and perhaps it would make it easier for many parents to open the subject if they realised what is the simple truth, that in most cases their children have observed many of the facts of life, although they do not know the laws underlying them. Where ignorance is really maintained it is often a real wrong to the child. By what other name than cowardice can we describe that reticence, calling itself modesty, in a mother, which allows a daughter to marry in utter ignorance of all that lies before her? It is really well for the girl if she is not as ignorant as her mother thinks her, though this does not excuse the mother for leaving to chance what ought to be taught of set purpose. It is true that many parents who feel their responsibility do not know how to begin to give the necessary knowledge. The difficulty thus felt has been realised and met by a special literature to supply the need. The Hon. Alfred Lyttelton, headmaster of Haileybury, has brought out a book on the instruction of the young in the laws of sex, and pamphlets meant to be given to the boys and girls themselves have been written by Mr. B. M'Call Barbour, Dr. S. Stall, Mrs. Wood-Allen, and Mrs. Drake. Parents who place these in the hands of their children are at least free from the reproach of that ostrich-like blindness which is often miscalled modesty.

### Appendicostomy.

In the *Medical News* of June 27th Dr. Willy Meyer gives an account of a patient on whom he had performed the operation, which was first suggested by Dr. Weir, of opening the appendix and making use of it for the purpose of a canal through which to apply local treatment to the large bowel in certain affections of that viscus. The proper designation for this operation in accordance with the rules of nomenclature, thinks Dr. Meyer, would be appendicostomy, and he names it accordingly. Apart from the name, to which no doubt sufficient objectors will be found, the procedure does not appear to be one that is altogether free from serious disadvantages. The appendix itself may be diseased, or adherent and difficult to find, and even when it is apparently normal its lumen may be quite occluded or so contracted as to prevent the insertion of a tube of sufficient size. Moreover, the patency cannot be determined until the organ has been opened. Another drawback is one that is mentioned by Dr. Meyer. In his case the tube, through which the bowel was flushed every other day, slipped out of the appendix, and it required long and tedious work to reforce an entry. A small valvular opening into the cæcum by Witzel's or Kader's method would afford an equally good means of applying local treatment to the large bowel without entailing any appreciable risk of the operation being a failure. For these reasons it is probable that appendicostomy will not find much favour with the majority of operating surgeons in this country. But as affording one more striking example of American ingenuity, if for no other reason, Dr. Meyer's account of his case of appendicostomy is of considerable interest.

